# Protracted exercise tolerance post-coronavirus disease 2019 in endurance athletes: A survey

**DOI:** 10.4102/sajp.v80i1.2063

**Published:** 2024-08-14

**Authors:** Cheryl A. Haley, Heleen van Aswegen, Elena Libhaber, Benita Olivier

**Affiliations:** 1 Department of Physiotherapy, Faculty of Health Sciences, University of the Witwatersrand, Johannesburg, South Africa; 2 Research Office, Faculty of Health Sciences, University of the Witwatersrand, Johannesburg, South Africa

**Keywords:** COVID-19, endurance athletes, return to sport, exercise capacity, fatigue

## Abstract

**Background:**

The global coronavirus disease 2019 (COVID-19) pandemic irrevocably influenced our lives, yet research in a diversity of countries is lacking. Cardiorespiratory fitness may be impaired for up to a year post-COVID-19 infection.

**Objectives:**

Our study aimed to compare acute and exertional symptoms, fatigue, and exercise performance in masters-age endurance athletes according to their return-to-sport status.

**Method:**

A cross-sectional survey-based observational study of long-distance runners and cyclists was conducted. Data were stratified into two groups: those who returned to their pre-illness level of sport and those who did not and were compared statistically.

**Results:**

A total of 308 survey responses were included in the analysis. The mean age of the athletes was 44.9 + 10.2 years, with 55.2% being male. The group that did not return to their pre-illness level of sport (31.5%) had more post-COVID sequelae, worse illness severity, with a higher frequency of resting and exertional symptoms, notably fatigue and dyspnoea. Decreased exercise capacity was correlated with increased physical fatigue scores.

**Conclusion:**

Almost one-third of endurance athletes suffered protracted exercise tolerance post-COVID-19. Long-term symptoms may be more consequential in this athlete population.

**Clinical Implications:**

Symptoms that may indicate cardiopulmonary consequences in recreational athletes should be investigated in order to facilitate return to sport and the important mental and physical benefits thereof. This will augment outcomes after respiratory tract infections and management of return to sport and expectations of endurance athletes.

## Introduction

The global coronavirus disease 2019 (COVID-19) pandemic, caused by the severe acute respiratory syndrome coronavirus-2 (SARS-CoV-2), has irrevocably influenced our lives. Extensive research has explored this disease and its devastating consequences on the world’s population and concurrently enhanced global knowledge of respiratory illnesses (Burgstahler et al. 2023). Comorbid diseases and a lack of regular physical activity predispose individuals to a more severe expression of COVID-19 (Chen et al. 2020; Snyders et al. 2022). However, high-intensity exercise may lower immune function and increase susceptibility to upper respiratory tract infections (Agha-Alinejad et al. 2022). Cardiorespiratory fitness is now classed as a vital sign (Mihalick et al. 2021), thus lagging athlete health and the hinderance of persistent symptoms in resuming pre-illness level of sport is worth assessing (Schroeder et al. 2023).

Coronavirus disease 2019 is a multifaceted disease with manifestations like no other virus, such as blood clotting and long-standing fatigue and dyspnoea (Schwellnus et al. 2021). The cardiovascular consequences of COVID-19 are by far the most threatening and range from peri-myocarditis, arrhythmias, thromboembolism, myocardial infarction, to sudden death (Monosilio et al. 2023). However, according to the American College of Cardiology (ACC) 2022 expert consensus statement, there is fortunately a low prevalence (Gluckman et al. 2022). Long-term respiratory sequelae are more likely post-COVID-19 (Durstenfeld et al. 2022). Pulmonary limitations have been found to be responsible for low exercise tolerance up to 1-year post-COVID-19 infection (Braga et al. 2023), even after mild illness (Vollrath et al. 2022).

Acute COVID-19 symptoms are categorised as asymptomatic, mild, moderate, severe, and critical illness according to the National Institute of Health (COVID-19 Treatment Guidelines 2023). Mild illness symptoms include malaise and/or fatigue, headache, myalgia, ageusia and/or anosmia, cough, fever, and diarrhoea and/or vomiting. Symptoms associated with moderate illness include dyspnoea and clinical evidence of lower respiratory disease on imaging with normal oxygen saturation (≥ 94% on room air). Individuals with severe illness present with respiratory distress and dyspnoea with oxygen saturation of < 94%, and critical illness includes respiratory failure, septic shock and multiorgan failure. In our study, the term long-COVID is used for new or persistent symptoms after 4 weeks post onset of illness as defined by the National Institute for Health and Care Excellence (NICE 2021). Fatigue is a multidimensional and subjective symptom that may or may not relate to other physiological factors (Smets et al. 1995).

During the early phase of the pandemic, most studies explored symptom severity and duration in collegiate elite athletes. There remains a paucity of literature concerning the masters athlete (over the age of 35 years) (Juhász et al. 2022). Endurance athletes require the ability to resist fatigue; typically training high volumes and placing a huge demand on their cardiorespiratory system (Joyner & Coyle 2008). To define and classify athletes, it has been proposed that competitive athletes exercise for 6 h or more per week and recreational athletes for at least 3 h; although endurance runners and cyclists often train equal volumes to professional athletes (pelliccia et al. 2021). Running is a highly accessible and affordable sport attracting long-distance adult athletes predominantly at a recreational level (DeJong, Fish & Hertel 2021). Unlike elite athletes, they may lack access to sports medicine professionals who disseminate return to sport (RTS) guidelines and screen for the risk of cardiac involvement (Elliott et al. 2020). Published RTS criteria are primarily based on the presence and severity of symptoms (Steinacker et al. 2022). This leads to concerns about adult recreational athletes exercising while potentially still feeling unwell (Ruuskanen et al. 2022). There is a need for prolonged observation and follow-up of athletes who had COVID-19, particularly with cardiovascular sequelae (Monosilio et al. 2023).

Our study aims to compare acute and exertional symptoms, fatigue, and exercise performance experienced by masters age group endurance athletes who had returned to their previous level of sport to those who had not. The results of our study may provide valuable insights for healthcare professionals, coaches, and athletes alike to understand variables associated with protracted RTS after COVID-19. This will facilitate better outcomes after respiratory tract infections and more effective management of RTS and expectations of competitive and recreational endurance athletes after respiratory tract infections.

## Research methods and design

### Study design

Our study is a cross-sectional survey-based observational to determine the difference in demographic and sport-specific characteristics, disease severity, duration, and symptoms, self-reported approach to RTS and exercise capacity, and fatigue score between athletes who had returned to their pre-illness level of sport and those who had not. The impetus for our study was the threat of COVID-19 consequences in athletes over the age of 35 years, doing a high volume of cardiovascular exercise. Participants were recruited by snowball sampling from athletic clubs in South Africa between December 2021 and July 2022. Emailers were sent to 64 athletic clubs containing the invitation to participate in the online survey and a request to forward it on to their members. The invitation was also disseminated via social media to 18 WhatsApp groups and 25 Facebook pages. Athletic clubs approved the post on their social media pages and chats. Invitations could then be forwarded by the recipients in a snowball fashion to fellow athletes.

### Study population and sampling strategy

Eligible participants were adult (age 18+) endurance athletes of both sexes who ran for three or more hours per week or cycled for three or more hours per week (McKinney et al. 2019). Those who participated in additional sports over and above were also included and described as multisport athletes. The athletes had either been diagnosed with COVID-19 through a positive polymerase chain reaction (PCR) test or exhibited the known symptoms of COVID-19. Athletes who were admitted to the intensive care unit (ICU) were excluded as ICU-acquired weakness occurs from 15–29 days, and necessitates prolonged rehabilitation to return to normal function (Van Aerde et al. 2020). Other exclusion criteria for athletes were not being proficient in the English language, residing outside of South Africa, and a lack of informed consent. The sample size estimation equalled 178. This was based on the change in aerobic capacity of the athletic population infected with COVID-19 compared to a control group (10.0% vs. 3.5%) (Crameri et al. 2020), with an effect size of 0.63, a confidence interval (CI) of 0.05, power of 0.9, and adding 10% for each confounding variable that is age, sex, sport, stage of disease. To achieve this sample size, we assumed a 30% response rate and 50 members per athletics club. At the time, the prevalence of COVID-19 infections in the city was 5.4%, thus, the questionnaire needed to be accessed by 3200 athletes at 64 clubs.

### Data collection

A self-administered web-based questionnaire was developed by the research team using the Research Electronic Data Capture (REDCap) platform and was based on current literature. Sociodemographic data including sex, age, and sport-specific data constituted the first 10 questions, followed by 10 questions on COVID-19 status, prevalence of comorbidities, and post-COVID sequelae. The third section included questions regarding the management of their acute illness based on the World Health Organization (WHO) Severity Scale (Marshall et al. 2020) and symptoms classified according to the National Institutes of Health (NIH) clinical spectrum (COVID-19 Treatment Guidelines, 2023). Symptom duration and time to return to their pre-illness level of sport were categorised to align with the NICE definitions (NICE 2021). The change in exercise tolerance was based on self-reported pace and power values.

Fatigue was scored using the standardised Multidimensional Fatigue Inventory (MDFI) tool (Smets et al. 1995). The MDFI consists of 20 questions that assess five categories of fatigue, namely: general fatigue, physical fatigue, reduced activity, reduced motivation, and mental fatigue. The scales used are freely available, thus no permissions were required. The MDFI has good internal consistency (Cronbach’s alpha = 0.84), and construct and convergent validity are reported (Smets et al. 1995). A professional panel in the field of sports medicine and respiratory health validated our study questionnaire. Construct validity of the questionnaire as a research tool was performed by sending a test questionnaire to 10 COVID-19 convalescent athletes, 4 of whom were medical professionals. Test-retest reliability was performed 2 weeks apart to avoid a learning effect. The null hypothesis of this study was that there was no difference in the effect of COVID-19 on the group that had returned to their pre-illness level of sports participation (y-RTS) versus those that had not (n-RTS). The primary outcome was a return to the pre-illness level of sport. Therefore, in the statistical analysis, we abbreviated the subgroup of athletes who selected the option ‘still not returned to pre-illness level of sport’ in the questionnaire as the n-RTS group. All others were combined into the y-RTS group.

### Data analysis

A lack of consent, answering ‘No’ to contracting COVID-19, or incomplete symptom data resulted in exclusion from data analysis. There was minimal missing data as radio button questions were marked *required. Data were extracted from REDCap into Microsoft Excel and imported into SPSS Statistics for Mac 28.0.1.1.(14) (IBM, Chicago, IL, United States [US]), which was used for all analyses. Descriptive characteristics were calculated using means and standard deviations for continuous variables, and percentages and frequencies for categorical variables for the total sample (All), the group that had returned to pre-illness level of sport (y-RTS), and the group that had not (n-RTS). The normality of distribution of continuous variables was assessed with the Shapiro–Wilk test. Independent t-tests were used to compare normally distributed variables and Mann–Whitney U test for parametric continuous data. Chi-square or Fisher’s exact tests were used to compare categorical data, with Cochran trend for ‘symptom duration’. Univariate and multivariate logistic regression was performed to evaluate the risk of not returning to pre-illness level of sport associated with the following variables: sex, age, general fatigue, physical fatigue, reduced activity, mental fatigue, reduced motivation, and exertional dyspnoea. Because of a strong association of general fatigue, physical fatigue and dyspnoea, only physical fatigue was included in the multivariate model. Model assumptions including goodness of fit, removal of residuals and collinearity were met.

### Ethical considerations

Unconditional ethical approval was provided by the University of the Witwatersrand’s Human Research Ethics Committee (Medical) (Certificate no. M210657). Our study procedures adhered to the principles set forth in the Declaration of Helsinki and the South African Guidelines for Good Clinical Practice and the Medical Research Council. An information sheet explaining our study purpose and data sharing according to the FAIR principles (GO FAIR, FAIR principles 2016) was linked to the survey and ensured confidentiality and anonymity and permission to withdraw from our study at any point. The participants were asked if they consented to the anonymised data being used for research purposes. After electronic consent was obtained, the full questionnaire opened. The completion of the questions implied that consent was given throughout. Only the research team had access to the REDCap platform on which the information sheet and survey was developed and data were stored. The exported data were stored one password-protected computer and backed up to Apple iCloud Drive.

## Results

### Demographic, sport-specific, and return to pre-illness level of sport characteristics of our study population

**FIGURE 1:**
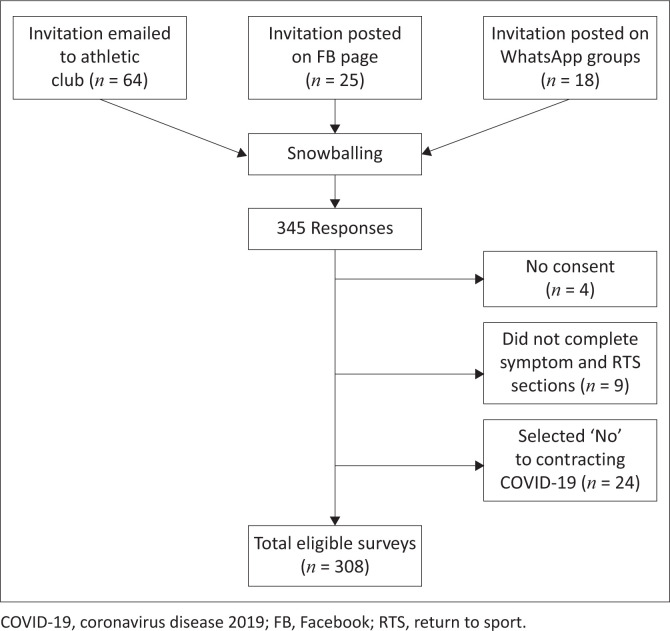
Study sample selection process.

After the surveys were assessed for eligibility, 308 participants were included in data analysis (Figure 1). Table 1 summarises our study population characteristics in three groups: all 308 participants, 211 (68.5%) who returned to pre-illness level of sport (y-RTS), and 97 (31.5%) who had not yet returned (n-RTS). The mean age was 44.9 + 10.2 years, and 170 (55.2%) were male. There is no significant difference in the demographic and sport-specific characteristics of y-RTS versus n-RTS.

**TABLE 1:** Demographic, sport-specific and coronavirus disease 2019 characteristics of our study population.

Variable	All (*N* = 308)		y-RTS (*n* = 211)		n-RTS (*n* = 97)		*p*
	*n*	%	*n*	%	*n*	%	
**Sex**	-	-	-	-	-	-	0.531
Male	170	55.2	119	56.4	51	52.6	-
Female	138	44.8	92	43.6	46	47.4	-
**Age groups (years)**	-	-	-	-	-	-	0.417
18–29	22	7.1	18	8.5	4	4.1	-
30–39	70	22.7	43	20.4	27	27.8	-
40–49	115	37.4	82	38.9	33	34.0	-
50–59	80	26.0	54	25.6	26	26.8	-
60–71	21	6.8	14	6.6	7	7.2	-
**Type of sport†**	-	-	-	-	-	-	0.598
Running	122	39.6	85	40.3	37	38.1	-
Cycling	55	17.9	40	19.0	15	15.5	-
Multisport	124	40.3	81	38.4	43	44.3	-
**Level of participation**	-	-	-	-	-	-	0.506
Elite	16	5.2	13	6.2	3	3.1	-
Competitive	99	32.1	66	31.3	33	34.0	-
Recreational	193	62.7	132	62.6	61	62.9	-
**Pre-COVID training volume (hours per week)**	-	-	-	-	-	-	0.503
< 6	84	30.3	63	29.8	21	21.7	-
≥ 6	224	72.7	148	70.1	76	78.4	-
**Comorbidities**	-	-	-	-	-	-	0.672
Yes	55	17.9	39	18.5	16	16.5	-
No	253	82.1	172	81.5	81	83.5	-
**Deteriorated pre-existing co-morbidities**	-	-	-	-	-	-	0.189
Yes	14	4.5	8	3.8	6	6.2	-
No	41	13.3	31	14.7	10	10.3	-
**Time from the onset of COVID-19 to study** ‡	-	-	-	-	-	-	0.151
≤ 3weeks	36	11.7	25	11.8	11	11.3	-
4–8 weeks	54	17.5	30	14.2	24	24.7	-
3–6 months	87	28.2	61	28.9	26	26.8	-
> 7 months	131	42.5	95	45.0	36	37.1	-
**Treatment WHO severity§**	-	-	-	-	-	-	0.032
0–2	158	51.3	117	55.5	41	42.3	-
3	150	48.7	94	44.5	56	57.7	-

COVID-19, coronavirus disease 2019; n-RTS, not returned to pre-illness level of sport; WHO, World Health Organization; y-RTS, returned to pre-illness level of sport.

† , type of sport was divided into ‘running’ = running only, ‘cycling’ = cycling only, and ‘multisport’ = additional sports including running or cycling.

‡ , we assume variant based on the elapsed time since onset of illness: omicron < 8 weeks, beta 3–6 months, delta > 7 months.

§ , World Health Organization COVID-19 Severity Scale: 0–2 = No treatment or did not consult a healthcare professional, 3 = Consulted a healthcare professional or admitted to hospital.

Most athletes did not have coexisting comorbidities (*n* = 253, 82.1%). However, comorbidities that were reported include asthma (*n* = 18, 5.8%), hypertension (*n* = 12, 3.8%), and cardiac disease (*n* = 4, 1.3%). The majority (94%) of athletes (*n* = 289) experienced one or more of the known COVID-19 symptoms. The only significant difference in COVID-19 characteristics of the y-RTS group compared to the n-RTS was the severity of the disease according to the WHO COVID-19 Severity Scale, indicating that more athletes consulted healthcare professionals and/or were admitted to hospital in the n-RTS group (*p* = 0.032).

### Acute and subacute symptoms

Table 2 summarises symptoms at the time of illness. There was no significant difference in asymptomatic disease experienced by the y-RTS versus n-RTS groups. A total of 21 participants (6.8%) tested negative, but their symptom presentation implied false negative tests. Fatigue was the only mild symptom more prevalent in the n-RTS group (*p* = 0.044). We found a higher proportion of moderate disease in n-RTS group (*p* = 0.006). There were eight athletes in total with severe illness requiring supplemental oxygen, with no significant difference between the groups (*p* = 0.710). No participants cited critical illness. Post-COVID-19 sequelae included cardiac (*n* = 15, 4.9%) and pulmonary (*n* = 12, 3.9%) consequences, and were more prevalent in the n-RTS group (*p* = 0.031).

**TABLE 2:** Coronavirus disease 2019 symptoms experienced at the time of illness.

Variable	All (*N* = 308)		y-RTS (*n* = 211, 68.5%)		n-RTS (*n* = 97, 31.5%)		*p*
	*n*	%	*n*	%	*n*	%	
**COVID-19**	-	-	-	-	-	-	0.201
symptoms	289	93.8	195	92.4	94	96.9	-
asymptomatic	19	6.2	16	7.6	3	3.1	-
**Acute symptoms†**							
Mild:							
Malaise or Fatigue	184	59.7	118	55.9	66	68.0	0.044
Headache	139	45.1	92	43.6	47	48.5	0.427
Myalgia	133	43.2	85	40.3	48	49.5	0.130
Ageusia or anosmia	84	27.3	56	26.5	41	42.3	0.670
Cough	83	26.9	51	24.2	32	33.0	0.105
Fever	63	20.5	42	19.9	21	21.6	0.724
Diarrhoea	21	21.6	9	4.3	3	3.1	0.445
Moderate:							
Dyspnoea SpO2 ≥ 94	97	31.5	56	26.5	41	42.3	0.006
Severe:							
Dyspnoea SpO2 ≤ 94	8	2.6	5	2.4	3	3.1	0.710
**Symptom duration (weeks)**	-	-	-	-	-	-	0.009
< 1	83	26.9	68	32.2	15	15.5	-
1–2	118	38.3	83	39.3	35	36.1	-
3–4	51	16.6	7	12.8	24	24.7	-
5–8	28	9.1	15	7.1	13	13.4	-
> 8	28	9.1	18	8.5	10	10.3	-
**Post-COVID-19 sequalae**	44	14.3	24	11.4	20	20.6	0.031

COVID-19, coronavirus disease 2019; n-RTS, not returned to pre-illness level of sport; WHO, World Health Organization; y-RTS, returned to pre-illness level of sport.

† , Multiple responses were allowed in the survey, resulting in cumulative percentages exceeding 100%. Symptoms are classified as mild, moderate, or severe illness according to the National Institute of Health (COVID-19 Treatment Guidelines).

### Factors associated with return to pre-illness level of sport

**FIGURE 2:**
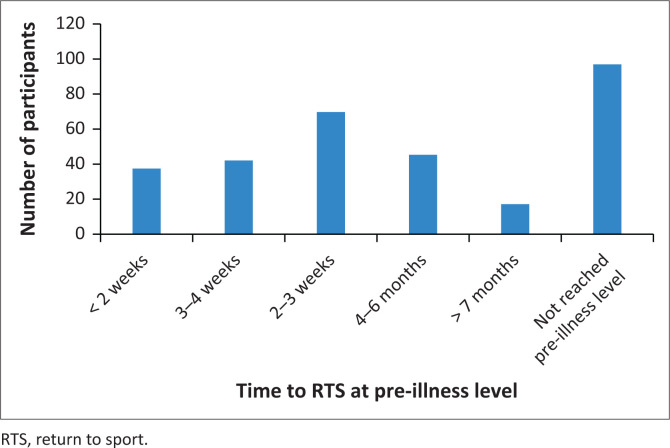
Time to return to pre-illness sport for our study population (*n* = 308).

The time frame in which athletes in the whole group returned to pre-illness level of sport is displayed in Figure 2, with *n* = 97 (31.5%) not yet at their previous level and constituted the n-RTS group. Table 3 displays the presence and severity of symptoms while exercising and other self-reported factors limiting their RTS. The athletes approached their resumption of training with similar consideration of most factors, with only pace being monitored more closely in the n-RTS group (*p* = 0.035). There was a distinctly higher proportion (*n* = 27, 27.8%) of athletes still experiencing symptoms at rest in the n-RTS group. The most frequently reported exertional symptoms by the whole group were fatigue (*n* = 216, 70.1%), and elevated heart rate (*n* = 216, 70.1%), followed by dyspnoea (*n* = 174, 56.5%). All symptoms except musculoskeletal pain occurred significantly more frequently in the n-RTS group. All self-reported limitations to exercise capacity were more common in the n-RTS group (*p* < 0.001). Training volume (*p* < 0.001), running pace (*p* < 0.001), and cycling threshold power (*p* = 0.024) were lower in the n-RTS group compared to the y-RTS group.

**TABLE 3:** The athlete’s approach to return to sport, the presence of exertional symptoms, challenges, and exercise capacity.

Variable	All (*N* = 308)				y-RTS (*n* = 211, 68.5%)				n-RTS (*n* = 97, 31.5%)				*p*
	*n*	%	Median	95% CI	*n*	%	Median	95% CI	*n*	%	Median	95% CI	
**Received RTS guidelines**	-	-	-	-	-	-	-	-	-	-	-	-	0.853
Yes	121	39.3	-	-	83	39.3	-	-	38	39.2	-	-	-
No	187	60.7	-	-	128	60.7	-	-	59	60.8	-	-	-
**Symptoms at rest**	-	-	-	-	-	-	-	-	-	-	-	-	0.005
Yes	56	18.2	-	-	29	13.7	-	-	27	27.8	-	-	-
No	252	81.8	-	-	182	86.3	-	-	70	72.2	-	-	-
**Monitoring during RTS**													
Resting, average, and maximal heart rate	215	69.8	-	-	143	67.8	-	-	72	74.2	-	-	0.252
Pace or speed	186	60.4	-	-	119	56.4	-	-	67	69.1	-	-	0.035
Rate of perceived exertion	100	32.5	-	-	71	33.6	-	-	29	29.9	-	-	0.514
Sleep quality	103	33.4	-	-	68	32.2	-	-	35	36.1	-	-	0.505
Stress	52	16.9	-	-	38	18.0	-	-	14	14.4	-	-	0.539
Fatigue	121	39.3	-	-	78	37.0	-	-	43	44.3	-	-	0.219
Myalgia	103	33.4	-	-	70	33.2	-	-	33	34.0	-	-	0.884
**Presence of symptoms while exercising†**													
Fatigue	216	70.1	-	-	134	63.4	-	-	82	84.6	-	-	< 0.001
Post-exertional malaise	183	59.4	-	-	105	49.8	-	-	78	80.4	-	-	< 0.001
Headache	105	34.2	-	-	61	29.0	-	-	44	45.4	-	-	0.005
Musculoskeletal pain	189	61.4	-	-	126	59.7	-	-	63	64.9	-	-	0.381
Autonomic disturbance including POTS‡	132	42.9	-	-	74	35.0	-	-	58	59.7	-	-	< 0.001
Elevated heart rate	216	70.0	-	-	131	62.1	-	-	85	87.6	-	-	< 0.001
Dyspnoea	174	56.5	-	-	104	49.3	-	-	70	72.2	-	-	< 0.001
Chest pain	64	20.7	-	-	30	14.2	-	-	34	35.0	-	-	< 0.001
**Self-reported factors limiting exercise tolerance**													
Poor endurance	199	64.6	-	-	108	51.2	-	-	91	93.8	-	-	< 0.001
Poor peak power	223	72.4	-	-	129	61.1	-	-	94	96.8	-	-	< 0.001
Difficulty hill climbing	220	71.4	-	-	133	63.0	-	-	87	89.7	-	-	< 0.001
Long-COVID-19 concerns	184	59.8	-	-	104	49.3	-	-	80	82.5	-	-	< 0.001
Anxiety or depression	147	47.7	-	-	82	38.9	-	-	65	67.0	-	-	< 0.001
Training volume pre-post§ (hours per week)	308	-	0.5	0.97–1.69	211	-	0.0	0.24–1.07	97	-	3.0	2.19–3.40	< 0.001
Running pace pre-post (min.km^-1^)	244	-	0.0	0.54–0.80	164	-	0.0	0.19–0.46	80	-	1.0	1.18–1.66	< 0.001
FTP pre-post (W.kg^-1^)	60	-	17.0	7.55–29.06	46	-	8.0	−4.62–22.68	14	-	30.0	17.53–61.87	0.024

CI, confidence interval; COVID-19, coronavirus disease 2019; FTP, Functional threshold power; n-RTS, not returned to pre-illness level of sport; RTS, return to sport; POTS, postural orthostatic tachycardia syndrome; y-RTS, returned to pre-illness level of sport.

† , Multiple responses were allowed in the survey, resulting in cumulative percentages exceeding 100%. Symptoms are classified as mild, moderate, or severe illness according to the National Institute of Health (COVID-19 Treatment Guidelines).

‡ , Data can fall in more than one category.

§ , Pre-post: Pre-COVID-19 minus post-COVID-19.

### Fatigue and the endurance athlete

The MDFI (Table 4) assesses five categories of fatigue (general fatigue, physical fatigue, reduced activity, reduced motivation and mental fatigue). Athletes who had not yet returned to pre-illness level scored higher in all fatigue categories, indicating considerably worse overall fatigue compared to the y-RTS group (*p* < 0.001). The results of the univariate regression analysis in Table 5 suggest all variables excluding age and sex are associated with RTS. The multivariate regression analysis adjusted for dyspnoea and general fatigue, showed that an increase in physical fatigue is associated with decreased odds of return to sport (OR 0.673, *p* < 0.001). An increase in mental fatigue is associated with increased odds of return to sport (OR 1.0361, *p* = 0.047).

**TABLE 4:** Multidimensional fatigue inventory scores.

Variable	All (*N* = 308) Mean ± s.d.	y-RTS (*n* = 211) Mean ± s.d.	n-RTS (*n* = 97) Mean ± s.d.	*p*
General fatigue	11.04 ± 3.99	9.70 ± 3.57	13.89 ± 3.28	< 0.001
Physical fatigue	9.48 ± 4.12	7.93 ± 3.49	12.79 ± 3.33	< 0.001
Reduced activity	8.36 ± 3.41	7.29 ± 2.86	10.63 ± 3.38	< 0.001
Reduced motivation	8.48 ± 3.45	7.58 ± 3.12	10.40 ± 3.34	< 0.001
Mental fatigue	10.11 ± 4.06	9.51 ± 4.06	11.37 ± 3.77	< 0.001
MDFI Total	47.44 ± 16.33	41.99 ± 14.40	59.08 ± 13.98	< 0.001

MDFI, multidimensional fatigue inventory; n-RTS, not returned to pre-illness level of sport; y-RTS, returned to pre-illness level of sport.

**TABLE 5:** Univariate and multivariate logistic regression analysis to determine the likelihood of variables explaining the variation in return to sport.

Variable	Univariate logistic regression			Multivariate logistic regression†		
	OR	95% CI	*p*	OR	95% CI	*p*
Age	0.995	0.971–1.019	0.672	1.019	0.988–1.052	0.222
Sex	0.857	0.529–1.389	0.531	1.311	0.696–2.470	0.402
General fatigue	0.781	0.659–0.783	< 0.001	-	-	-
Physical fatigue	0.702	0.645–0.764	< 0.001	0.673	0.592–0.766	< 0.001
Reduced activity	0.724	0.662–0.791	< 0.001	0.884	0.756–1.034	0.123
Mental fatigue	0.891	0.837–0.948	< 0.001	1.036	1.001–1.223	0.047
Reduced motivation	0.777	0.781–0.841	< 0.001	1.107	0.898–1.194	0.632
Dyspnoea	0.375	0.223–0.630	< 0.001	-	-	-

OR, odds ratio; CI, confidence interval.

† , Hosmer and Lemershow goodness-of-fit test *p* = 0.495.

## Discussion

Our study provides insight into acute and exertional symptoms associated with protracted exercise tolerance in a masters-age athlete population that does a high volume of cardiovascular exercise yet has had little investigation. Following several studies earlier in the pandemic that focussed on elite team sport athletes (Petek et al. 2021), our study included a large sample of endurance athletes, closely matching the demographics of marathon runners worldwide. Because of a global shortage of polymerase chain reaction (PCR) tests in 2020, and the questionable reliability thereof (Burgstahler et al. 2023), many diagnoses were based on symptom presentation or positive contact. Therefore, studies such as ours included symptomatic individuals who may not have been tested or had either a positive or negative PCR test result. Our findings suggest that acute fatigue, longer symptom duration, post-COVID-19 sequelae, exertional symptoms especially dyspnoea, and chronic physical fatigue are associated with protracted RTS in endurance athletes.

Nearly one third of the athletes had not yet returned to pre-illness levels at the time of data collection. Many (27.8%) were still experiencing symptoms at rest, potentially explaining the less than 6-h per week training volume typical of recreational endurance athletes (McKinney et al. 2019). The most frequently reported acute symptoms in our study (fatigue, headache, and myalgia) were consistent with the findings of other studies (Lopes et al. 2021; Magnusson et al. 2022; Sewry et al. 2022), yet only fatigue was associated with protracted RTS in our study. Athletes have missed training more often because of fatigue after COVID-19 than any other viral illnesses or symptoms (Schwellnus et al. 2021).

The presence of exertional symptoms is widely documented, suggesting that athletes begin training while feeling unwell (Schroeder et al. 2023). Our study confirmed this, as 70% of the entire group experienced fatigue or elevated heart rate while exercising, 57% experienced dyspnoea, and 21% experienced chest pain. Hughes et al. (2022) proposed no exercise resumption until symptoms had abated or diminished. Whether the 35% of the n-RTS group who experienced chest pain followed RTS guidelines and thereby reduced their training is unknown, as 14% of the y-RTS group also reported chest pain. Although the reasons why the athletes did not return to pre-illness level was not specifically investigated, the results suggest that persistent symptoms interfered with full athletic performance.

In our study, athletes with post-COVID-19 sequelae were less likely to return to their previous performance levels, consistent with Schroeder et al. (2023). Our study found a higher prevalence of cardiovascular sequelae (8.8%) compared to those reviewed by Monosilio et al. (2023), probably because of this study’s higher average age. Herein lies an important finding. Myocarditis, arrythmias and other cardiopulmonary post-COVID-19 sequelae presented in our study, are a concern and may be underreported in masters age-group athletes. Ventricular arrhythmias were found to occur in asymptomatic or mild disease (Casasco et al. 2022), and these athletes, constituting the bulk of the field in marathons events, may not have undergone routine cardiac screening. Our findings imply that older athletes and those with post-COVID-19 sequelae require better management. These athletes should monitor resting and exertional heart rate, sleep quality, cardiopulmonary symptoms, and fatigue after resuming exercise (Steinacker et al. 2022). While the athletes in our study were adept at monitoring heart rate and pace facilitated by popular wearable devices, it is recommended that athletes be more aware of the symptoms they experience and the implications thereof on RTS.

A novel finding in our study was the significantly decreased odds of RTS in those with physical fatigue, while mental fatigue is strongly associated with increased odds of RTS. This may seem counterintuitive as fatigue and impaired neurocognitive ability can lead to protracted exercise tolerance (Steinacker et al. 2022). Mental fatigue often occurs because of a prolonged period of demanding cognitive tasks yet may also develop from the frustration of trying harder to resume fitness compared to other respiratory illnesses (Marcora et al. 2009). In a scoping review by Oswald et al. (2020), running has been unequivocally positively associated with improving mood, sense of well-being, self-efficacy, and decreased depression. Regular runners who were prevented from running for 2 weeks had increased anxiety and depression compared to the runners who continued (Oswald et al. 2020). Hence, an unexpected finding becomes expected. This relationship may further explain the tendency for endurance athletes to resume training before recovering sufficiently.

Our study is one of the first to compare individuals who successfully rebuilt their pre-illness fitness level to those who were still struggling. Our results suggest that the n-RTS group intentionally reduced the intensity of their training during recovery. Post-COVID-19 performance capacity as determined by training volume, running speed, and cycling threshold power was lower in the n-RTS group when compared to pre-COVID-19 levels. In contrast, Emeran et al. (2022) found no difference in running speed or training volume. A large proportion of athletes did not return to sport after having moderate illness, likely because of the symptom severity directly precluding RTS, or recommendations from healthcare providers (Schroeder et al. 2023), as more consultations were attended by athletes in the n-RTS group. Longer symptom duration, and especially persistent fatigue was more prevalent in the n-RTS group, which can negatively affect quality of life (Vollrath et al. 2022). As research investigating long-term consequences of COVID-19 on cardiorespiratory fitness and sports participation emerges, the knowledge gained needs to be disseminated to athletes, coaches, and medical professionals.

### Strengths and limitations

The main strength of this study is the sample size and statistical power. Athletes were recruited throughout South Africa, which enhances generalisability to the South African athletic population, although generalisation may not be translated to other countries. The focus of our study was endurance athletes and therefore mostly older than 35 years of age, fulfilling a gap in the literature. Quantifying subjective descriptions of fatigue by using the MDFI is a strength of our study as to our knowledge, it has not been used in other studies on athletic populations. Future studies using the MDFI in athletes is recommended. This is one of few studies that has associated exertional symptoms with RTS status.

As is customary for surveys, certain limitations exist. Snowball sampling will not allow a response rate to be calculated and recorded, thus reducing the power of our study. The participant’s responses rely on retrospective recall (recall bias) and the period from illness to the time of our study will vary between participants. The inclusion criteria did not specify the presence of COVID-19 symptoms, thus selection bias caused underrepresentation of the number of asymptomatic cases. Vaccine status was unknown; however, by deducing from the time of illness, most athletes would not have had the opportunity to be vaccinated. Athletes in our study would have been infected by a mix of viral strains. We did not ask whether home oxygen was used, which may have placed some participants not admitted to the hospital in the ‘severe illness’ category. These limitations were unavoidable with the current knowledge at the time of our study. No direct cause-effect relationship can be determined from our observational study.

### Interpretation

These findings may provide valuable insights into the acute and long-term manifestations of this disease in a masters-age athlete population. Symptoms that may indicate cardiopulmonary consequences in an athlete population that does a high volume of cardiovascular exercise should be investigated. An interdisciplinary approach to management is vital to facilitate return to sport and the important mental and physical benefits thereof. This will augment outcomes after respiratory tract infections in endurance athletes.

## Conclusion

Our findings suggest that acute fatigue, longer symptom duration, post-COVID sequelae, the presence of exertional symptoms particularly dyspnoea, and chronic physical fatigue are associated with protracted RTS in endurance athletes. These symptoms still abound albeit less frequently after infection by the current Omicron variant. Such symptoms should continue to be flagged and managed to prevent protracted RTS, with further investigations if cardiopulmonary consequences are suspected. Consideration of the mental gains of physical activity ought to be given high regard while building the endurance athlete’s training load to return to their pre-illness level.
